# Tuberculosis

**Published:** 1920-10-16

**Authors:** 


THE PUBLIC WELL-BEING—(*continued*).

### Tuberculosis.

### THE WIDER DIFFICULTIES OF TREATMENT.

There are certain broad facts which must always be kept in mind when the scientific fight against disease is considered. The technical methods may be sound, but there may often be—as there often are—conditions of life, over which medical scientists have no control, handicapping every step they take. Tuberculosis is a case in point. The National Association for the Prevention and Cure of Consumption, which met in Liverpool last week, (and is referred to on page 51) had to lament the comparative want of success of sanatorium treatment; but most of the contributors to the debate showed that the best use had not been made of sanatoria, owing to general circumstances of life that prevented sufficiently long residence of patients and that took them back after treatment into unsuitable surroundings. Much, first of all, depends on the kind of case admitted for treatment, but, as Dr. Nathan Raw, M.P., asserted, it is not possible to get satisfactory results if the patient is unable to devote sufficient time to his cure, and, even if he can do so, the benefits are largely nullified by patients having to return to unsanitary home conditions and unsuitable conditions of work. This is the difficult side of the question. If consumptives could be segregated and given suitable work in proper circumstances for their well-being it would be comparatively simple. But it is not a possible solution, and the solution must be looked for in finding some modified form of work and in generally increasing the standard of existence. Poverty, bad housing, bad food, and improper clothing must always be agents in the spread of the disease, and the doctors have no fair chance against them. Mr. Stewart, of Glasgow, attributed the recent decrease in tuberculosis to the fact that poverty, so far as the supply of food and clothing was concerned, had greatly diminished. It may be so, but people have still much to learn in the right expenditure of money upon wholesome foods.

The Conference also dismissed the relation of milk to tuberculosis, especially in reference to children. Some members actually advocated less consumption of milk because of its power of infection, but Professor J. M. Beattie claimed that the proprietary milk preparations for feeding children were not practical substitutes for fresh milk. Sterilisation and pasteurisation were not guarantees against tubercle infection; milk must be dealt with at the sources of supply, with special precautions against tuberculous animals. Dr. Macfadden, of the Ministry of Health, spoke of the necessity for increasing largely the consumption of fresh milk and for improving its quality; he suggested that public health authorities should combine with their direct endeavours to protect the community from infected milk an active policy for increasing its consumption. Whatever is done, it is urgent that definite and decisive steps should be taken somehow to secure greater purity in milk. The repeated emphasis of the dangers of infected milk without corresponding and obvious measures to improve it is not calculated to further the cause of fresh milk as an important food. Progress has undoubtedly been made, and there are greater opportunities to get a purer supply. But it must become far more complete and far more general.

